# COPD association and repeatability of blood biomarkers in the ECLIPSE cohort

**DOI:** 10.1186/1465-9921-12-146

**Published:** 2011-11-04

**Authors:** Jennifer A Dickens, Bruce E Miller, Lisa D Edwards, Edwin K Silverman, David A Lomas, Ruth Tal-Singer

**Affiliations:** 1 Department of Medicine, University of Cambridge, Cambridge Institute for Medical Research, Cambridge, UK; 2 GlaxoSmithKline, King of Prussia, PA, USA; 3 GlaxoSmithKline, Research Triangle Park, NC, USA; 4 The Channing Laboratory and Pulmonary and Critical Care Division, Brigham and Women's Hospital and Harvard Medical School, Boston, Massachusetts, USA

**Keywords:** Biomarkers, Chronic Obstructive Pulmonary Disease (COPD), Evaluation of COPD Longitudinally to Identify Surrogate Endpoints (ECLIPSE), Inflammation

## Abstract

**Background:**

There is a need for biomarkers to better characterise individuals with COPD and to aid with the development of therapeutic interventions. A panel of putative blood biomarkers was assessed in a subgroup of the Evaluation of COPD Longitudinally to Identify Surrogate Endpoints (ECLIPSE) cohort.

**Methods:**

Thirty-four blood biomarkers were assessed in 201 subjects with COPD, 37 ex-smoker controls with normal lung function and 37 healthy non-smokers selected from the ECLIPSE cohort. Biomarker repeatability was assessed using baseline and 3-month samples. Intergroup comparisons were made using analysis of variance, repeatability was assessed through Bland-Altman plots, and correlations between biomarkers and clinical characteristics were assessed using Spearman correlation coefficients.

**Results:**

Fifteen biomarkers were significantly different in individuals with COPD when compared to former or non-smoker controls. Some biomarkers, including tumor necrosis factor-α and interferon-γ, were measurable in only a minority of subjects whilst others such as C-reactive protein showed wide variability over the 3-month replication period. Fibrinogen was the most repeatable biomarker and exhibited a weak correlation with 6-minute walk distance, exacerbation rate, BODE index and MRC dyspnoea score in COPD subjects. 33% (66/201) of the COPD subjects reported at least 1 exacerbation over the 3 month study with 18% (36/201) reporting the exacerbation within 30 days of the 3-month visit. CRP, fibrinogen interleukin-6 and surfactant protein-D were significantly elevated in those COPD subjects with exacerbations within 30 days of the 3-month visit compared with those individuals that did not exacerbate or whose exacerbations had resolved.

**Conclusions:**

Only a few of the biomarkers assessed may be useful in diagnosis or management of COPD where the diagnosis is based on airflow obstruction (GOLD). Further analysis of more promising biomarkers may reveal utility in subsets of patients. Fibrinogen in particular has emerged as a potentially useful biomarker from this cohort and requires further investigation.

**Trial Registration:**

SCO104960, clinicaltrials.gov identifier NCT00292552

## Background

Chronic obstructive pulmonary disease (COPD) is a major cause of global morbidity and mortality and is predicted to become the third leading cause of death by 2020 [1]. It is a multicomponent condition involving both local and systemic pathological processes that include airway obstruction, emphysema, mucus hypersecretion, loss of lean body mass and an increased risk of cardiovascular disease [2]. The most widely used marker of disease severity and progression is the forced expiratory volume in one second (FEV_1_). However FEV_1 _correlates poorly with both symptoms and other measures of disease progression [3]. Moreover it does not differentiate between the causes of airflow obstruction (i.e. small airways disease or emphysema) or identify the extra-pulmonary manifestations of COPD. There is clearly a need for biomarkers that can aid with the diagnosis, risk stratification and the assessment of therapeutic interventions. The search for biomarkers has centred around proteins and other molecules in exhaled breath condensate, sputum, urine, bronchoalveolar lavage and blood that have been implicated in the pathogenesis of COPD [4]. Profiling of blood biomarkers has identified a number of biomarkers that may distinguish individuals with COPD from control subjects [5]. However, additional research is needed to determine the value of biomarkers in characterizing COPD patients, following disease progression, and/or the ability to serve as intermediate or surrogate endpoints in clinical studies.

We have used the ECLIPSE (Evaluation of COPD Longitudinally to Identify Surrogate Endpoints) cohort to evaluate a number of previously reported biomarkers, and to identify new biomarkers for COPD [6]. This cohort has already been used to evaluate serum CC-16, surfactant protein D (SP-D) and CCL-18/PARC. SP-D and CCL-18/PARC were elevated and CC-16 was decreased in individuals with COPD when compared to control subjects [7-9]. We report here the assessment of a panel of 34 biomarkers in a subgroup of individuals from the ECLIPSE cohort. The biomarkers were selected based on previous work suggesting their potential association with the pathogenesis of COPD [5,10].

## Methods

A sub-group of individuals was selected from the ECLIPSE study (SCO104960, Clinicaltrials.gov identifier NCT00292552) as the "biomarker cohort" in order to evaluate 34 potential blood biomarkers in COPD [7,8]. The aims and operational aspects of ECLIPSE have been described elsewhere [6]. Briefly ECLIPSE is a 3-year multicentre, longitudinal, prospective study to identify novel endpoints in COPD. Individuals aged 40-75 years were recruited to the study if they had a smoking history of ≥ 10 pack-years, a post bronchodilator ratio between forced expiratory volume in 1 second (FEV_1_) and forced vital capacity (FVC) ≤ 0.7 and Global Initiative on Obstructive Lung Disease (GOLD) stage II (FEV_1 _50-80% predicted), III (FEV_1 _30-50% predicted) or IV (FEV_1 _< 30% predicted) COPD. Smoking (≥ 10 pack-years) and non-smoking (< 1 pack-year) control subjects were enrolled if they were aged 40-75 years and had normal lung function (post bronchodilator FEV_1 _> 85% predicted and FEV_1_/FVC ratio > 0.7). The "biomarker cohort" consisted of 275 subjects (201 COPD subjects with GOLD stage II-IV disease, 37 ex-smoker controls and 37 non-smoker controls) matched to the full ECLIPSE cohort. Only former smokers were included to avoid the influence of acute cigarette smoke exposure that might increase variability in our relatively small sample size.

The methods used to evaluate lung function and the extent of emphysema have been described previously [8]. The number of exacerbations was assessed by telephone contact on a monthly basis enquiring about details of contact with doctors or hospital and the need to take medications for exacerbations (oral steroids and/or antibiotics) [11]. Exercise capacity was measured at enrolment with a standard supervised six-minute walk test. Assessments of depression, fatigue and level of dyspnoea were assessed with the CES-D (centre of epidemiological studies depression scale) [12], FACIT (functional assessment of chronic illness therapy) [13] and modified MRC (Medical Research Council) [14] dyspnoea scores, respectively.

### Measurement of blood biomarkers

Whole blood was collected into vacutainer tubes at the timepoints specified. For serum preparation, the blood was allowed to clot for 30 minutes and serum was obtained by centrifugation at 1500 g for 10-15 minutes. For plasma preparation, whole blood was collected into vacutainer tubes containing EDTA. Plasma was obtained by centrifugation at 2000 g for 10-15 minutes. Serum and plasma samples were stored at -80°C until analysed. With the exception of fibrinogen and adiponectin, all biomarkers were measured using validated immunoassays on the SearchLight Protein Array Platform (Aushon Biosystems, Inc., Billerica, MA USA). Fibrinogen was measured using an immunoturbidometric assay validated for use with EDTA plasma (K-ASSAY fibrinogen test, Kamiya Biomedical Co., Seattle, WA, USA). Adiponectin was measured using an individual ELISA method (Linco Research, St. Charles, MO, USA). SP-D and CC-16 concentrations were measured in serum as described previously [7,8]. Assays were performed in duplicate to allow assessment of assay variation (Additional File 1).

Repeatability over two visits was assessed by measuring biomarker levels at baseline and at 3 months. The analysis included all subjects that had appropriate samples at the respective timepoints. In addition, 5 serum biomarkers were assessed at a single timepoint at the 6 months study visit: adiponectin, β-defensin-2, CXCL7 (chemokine (C-X-C motif) ligand 7), leptin and MMP-8 (matrix metalloproteinase 8). Additional details of the assay methods as well as assay performance information are available in additional file 1.

### Statistical Analysis

Variability of analytes was assessed through Bland-Altman plots and frequency histograms. Due to non-normality of biomarker results as identified by Shapiro-Wilk and Kolmogorov-Smirnov tests, all values in the biomarker cohort were log-transformed prior to analysis. All comparisons between groups were then conducted by analysis of variance (ANOVA) based on the log-transformed values with adjustment made for differences in age and sex seen between the three groups. Spearman correlation coefficients (based on ranks) were calculated for correlations between biomarkers and clinical parameters. Analyte results that were below the limit of quantification (LLOQ) were imputed as 1/2 times the LLOQ. All analyses were performed with SAS^® ^Version 9.1 (SAS Institute, Cary, NC). Differences were considered significant if p ≤ 0.05. No adjustments were made for multiple comparisons.

### Ethics

The ECLIPSE study was conducted in accordance with the Declaration of Helsinki and ICH Good Clinical Practice Guidelines. The study protocol was approved by the local ethics committees for all 46 participating sites in 12 countries. All participants gave written informed consent.

## Results

### Assessment of biomarkers in COPD and control subjects

The characteristics of the cohort selected for the biomarker analysis are shown in Table 1. The cohort consisted of 201 individuals (all former smokers) with GOLD stage II-IV COPD, 37 smoker controls and 37 non-smoker controls. There were fewer males in the non-smoker control group and individuals in both control groups were on average younger than COPD subjects; multivariate analysis was used to adjust for age and sex prior to comparison of data between groups. Comparisons were made between potential biomarkers in COPD subjects and both smoker and non-smoker controls with normal lung function. At baseline, 15 analytes were significantly different between COPD subjects and either smoker or non-smoker controls: adiponectin, β-defensin-2, CRP (C-reactive protein), CCL18 (Chemokine (C-C motif) ligand 18), fibrinogen, hepatocyte growth factor, CXCL10, IL-12p40, IL-6, IL-8, MMP-8, MMP-9, CCL2, myeloperoxidase and prolactin (Table 2). Significant differences between individuals with COPD and both control groups were seen with β-defensin-2, CRP, CCL18, fibrinogen and MMP-8.

**Table 1:** Baseline characteristics of the biomarker cohort.

Characteristic		COPD Subjects	Smoker Controls	Non-smoker Controls
Number		201	37	37

Age (yrs)		64.5 (6.0)	60.7 (7.6)	60.0 (8.8)

Male (%)		73%	68%	38%

Smoking, pack-years		45.7 (26.9)	29.3 (16.5)	0.0 (0.2)

FEV_1_ % predicted		43.8 (17.2)	109.6 (12.3)	115.9 (11.9)

% Low attenuation area on CT (< -950HU)		22.7 (13.7)	4.6 (4.5)	5.3 (5.5)

Radiologist Score	< 5% emphysema	20%	95%	97%

	5-25% emphysema	18%	5%	0

	25-50% emphysema	15%	0	3%

	> 50% emphysema	48%	0	0

% reversibility FEV_1_		9.6 (11.2)	3.7 (5.1)	2.2 (4.9)

BMI		26.9 (5.5)	29.2 (4.9)	27.1 (6.1)

Fat free mass index		17.1 (2.6)	18.2 (2.5)	16.8 (2.6)

Number of exacerbations in year prior to enrollment in ECLIPSE	0	46%	100%	97%
	
	1	26%	0	3%
	
	2	18%	0	0
	
	3 or more	10%	0	0

6 min walk (m)		384 (124)		

BODE Index		3.4 (2.2)		

mMRC score		1.9 (1.1)	0.2 (0.5)	0.1 (0.4)

CES-D score		11.5 (9.0)	7.1 (8.8)	5.3 (5.1)

FACIT score		34.8 (10.6)	44.4 (8.4)	47.4 (4.3)

SGRQ-C Total score		52.9 (18.9)	8.4 (11.9)	2.5 (2.3)

BMI = Body Mass Index; BODE = BMI (B), airflow obstruction (O) as measured by the post-bronchodilator FEV_1 _(percentage of predicted value), dyspnoea (D) assessed by the modified Medical Research Council (mMRC) score, and exercise tolerance (E) measured by 6-minute walking distance; FEV_1_ = Forced Expiratory Volume in 1 second; SGRQ-C = St. Georges respiratory questionnaire for COPD patients. Values expressed as mean (standard deviation) or as percentage.

**Table 2:** Baseline values for biomarkers with a significant difference between COPD subjects and either smoking or non-smoking controls.

	COPD Subjects		Smoker Controls			Non-smoker Controls		
**Biomarker**	**N**	**Median (IQR)**	**N**	**Median (IQR)**	**COPD Subjects vs Smoker Controls**	**N**	**Median (IQR)**	**COPD Subjects vs Non-smoker Controls**

Adiponectin (ng/mL)*	198	15614.5 (10340.0)	37	10856.0 (6145.0)	**< 0.001**	36	16124.5 (6428.5)	0.270

β-Defensin-2 (pg/mL)*	199	1222.3 (1794.8)	37	771.6 (784.2)	**0.003**	37	648.2 (964.1)	**0.042**

CCL2 (pg/mL)	201	615.0 (395.0)	37	530.0 (365.0)	0.065	37	460.0 (445.0)	**0.017**

CCL18 (pg/mL)	193	96000.0 (93000.0)	37	67000.0 (40000.0)	**0.018**	35	68000.0 (34000.0)	**0.041**

C-reactive protein (μg/mL)	200	5.38 (10.62)	37	3.13 (5.27)	**0.036**	35	2.17 (3.96)	**0.002**

CXCL10 (pg/mL)	198	79.7 (38.0)	37	67.8 (26.6)	0.085	35	65.8 (30.4)	**0.017**

Fibrinogen (mg/dL)	200	466.0 (117.5)	37	425.0 (100.0)	**0.002**	37	387.0 (83.0)	**< 0.001**

Hepatocyte growth factor (pg/mL)	199	589.2 (492.7)	37	475.3 (303.6)	0.324	37	421.7 (217.7)	**0.012**

Interleukin-6 (pg/mL)	194	0.2 (2.8)	37	0.2 (0.0)	**0.004**	37	0.2 (0.4)	0.072

Interleukin-8 (pg/mL)	190	8.8 (5.2)	36	7.7 (4.8)	0.051	34	6.7 (3.0)	**0.009**

Interleukin-12 p40 (pg/mL)	177	1.6 (2.8)	32	2.3 (3.5)	0.343	32	0.6 (2.3)	**0.017**

Matrix metalloproteinase-8 (pg/mL)*	198	9005.8 (10853.0)	37	7393.7 (7231.2)	**0.003**	37	6607.9 (5241.8)	**< 0.001**

Matrix metalloproteinase-9 (pg/mL)	201	238020.0 (202960.0)	37	182700.0 (92460.0)	0.198	37	158180.0 (85815.0)	**< 0.001**

Myeloperoxidase (pg/mL)	198	34680.0 (34305.0)	36	24770.0 (19280.0)	0.195	37	20800.0 (17100.0)	**0.043**

Prolactin (pg/mL)	196	525.0 (462.5)	37	345.0 (260.0)	**< 0.001**	36	580.0 (325.0)	0.771

Values are expressed as median (IQR), p values are based on log transformed results adjusted for age and sex. Biomarkers marked with * were measured at a single timepoint.

There were no significant differences between cases and controls for the other markers and therefore these were not analysed further (Table 3). Importantly, the concentrations of a number of analytes were above the lower limit of quantification in only a small percentage of subjects: TNF-α (10% COPD subjects, 10% combined controls), interferon-γ (6% COPD subjects, 6% combined controls), IL-10 (14% COPD subjects, 12% combined controls), IL-15 (17% COPD subjects, 15% combined controls), IL-17 (< 1% COPD subjects, 0% combined controls) and IL-1β (21% COPD subjects, 22% combined controls).

**Table 3:** Baseline values for biomarkers in which there was no significant difference between COPD subjects and either smoking or non-smoking controls.

	COPD Subjects		Smoker Controls			Non-smoker Controls		
**Biomarker**	**N**	**Median (IQR)**	**N**	**Median (IQR)**	**COPD Subjects vs Smoker Controls**	**N**	**Median (IQR)**	**COPD Subjects vs Non-smoker Controls**

Brain-derived neurotrophic growth factor (pg/mL)	200	36640.0 (17870.0)	37	35140.0 (14420.0)	0.656	37	35620.0 (10060.0)	0.381

CCL4 (pg/mL)	201	109.8 (86.0)	37	117.2 (70.0)	0.846	37	89.6 (86.2)	0.183

CCL23 (pg/mL)	201	501.8 (217.0)	37	443.0 (210.6)	0.483	37	468.0 (200.4)	0.178

CCL24 (pg/mL)	193	93.5 (124.0)	36	74.5 (100.0)	0.294	35	60.0 (61.5)	0.089

CXCL5 (pg/mL)	201	1415.0 (1065.0)	37	1265.0 (500.0)	0.144	37	1425.0 (975.0)	0.817

CXCL7 (pg/mL)*	199	34270367 (21359723)	37	36867340 (17597022)	0.582	36	33070725 (16762456)	0.425

CXCL11 (pg/mL)	200	19.8 (15.4)	36	18.4 (17.3)	0.488	36	16.8 (13.3)	0.249

Interferon-γ (pg/mL)	201	0.4 (0.0)	37	0.4 (0.0)	0.346	35	0.4 (0.0)	0.584

Interleukin-1β (pg/mL)	199	0.2 (0.0)	36	0.2 (0.2)	0.185	37	0.2 (0.0)	0.576

Interleukin-10 (pg/mL)	201	0.4 (0.0)	37	0.4 (0.0)	0.697	37	0.4 (0.0)	0.433

Interleukin-15 (pg/mL)	187	0.8 (0.0)	37	0.8 (0.0)	0.227	36	0.8 (0.0)	0.971

Interleukin-17 (pg/mL)	201	0.8 (0.0)	37	0.8 (0.0)	0.630	37	0.8 (0.0)	0.632

Interleukin-1 receptor antagonist (pg/mL)	193	217.4 (269.9)	36	204.6 (127.6)	0.609	34	139.0 (130.0)	0.056

Leptin (pg/mL)*	199	12857.5 (21025.5)	37	10074.8 (16770.9)	0.622	37	13103.6 (26107.2)	0.058

Tissue inhibitor of metalloproteinase-1 (pg/mL)	201	329500.0 (142100.0)	37	285800.0 (128900.0)	0.215	37	255100.0 (149400.0)	0.302

Transforming Growth Factor-α (pg/mL)	197	3.9 (10.0)	36	3.7 (9.7)	0.674	36	1.2 (5.4)	0.056

Tumor necrosis factor receptor type I (pg/mL)	201	1530.0 (655.0)	37	1525.0 (521.4)	0.999	37	1400.0 (600.0)	0.494

Tumor necrosis factor receptor type II (pg/mL)	196	1065.0 (545.0)	37	920.0 (525.0)	0.454	36	915.0 (537.5)	0.930

Tumor necrosis factor-α (pg/mL)	201	2.4 (0.0)	36	2.4 (0.0)	0.178	37	2.4 (0.0)	0.667

Values expressed as median (IQR), p values based on log transformed results adjusted for age and sex. Biomarkers marked with * were measured at a single timepoint.

### Assessment of biomarkers at 3 months and the effect of exacerbations

Over the 3 month period of this study, 33% (66/201) of the COPD subjects reported at least one exacerbation as defined by the requirement for antibiotics or oral corticosteroids or admission to hospital. Of these, 18% (36/201) of the COPD subjects had exacerbations within 30 days of the 3-month study visit. In addition, 4 COPD subjects were still taking an oral steroid within 2 weeks of the 3-month visit and 1 subject was taking azithromycin. CRP, fibrinogen, IL-6 and SP-D were significantly higher in these subjects (Table 4 and Additional File 2).

**Table 4:** The effect of exacerbations on biomarkers.

Biomarker	Exacerbation within 30 days		No or Resolved Exacerbations		p-value
	
	N	Median (IQR)	N	Median (IQR)	
Fibrinogen (mg/dL)	33	534.0 (156.0)	157	464.0 (115.0)	< 0.001

Interleukin-6 (pg/mL)	30	2.8 (6.0)	151	0.6 (2.6)	< 0.001

Surfactant Protein D (ng/mL)	32	132.7 (135.9)	148	101.0 (70.7)	0.011

C-reactive protein (μg/mL)	33	6.96 (6.52)	153	3.98 (6.76)	0.048

Biomarker results at 3 months for COPD subjects with an exacerbation within 30 days of the study visit and COPD subjects with no exacerbations or resolved exacerbations. Values are expressed as median (IQR), p values are based on log transformed results. The table shows those biomarkers that were significantly different between the two groups. Results for the remaining biomarkers are included additional Table 2.

### Assessment of repeatability over 3 months

**Figure 1:**
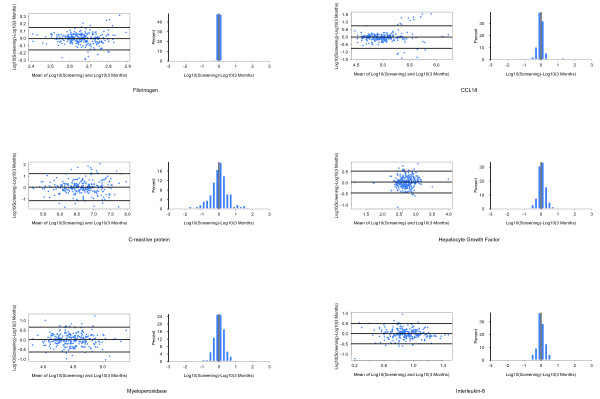
**Bland Altman plots and frequency histograms of differences between baseline and 3-month results illustrating variability of selected biomarkers**. All values have been log transformed prior to analysis.

Twelve of the 15 biomarkers of interest were assessed for repeatability between the baseline and 3-month study visits. Results from subjects with either exacerbations within 4 weeks or who had been taking oral steroids within 2 weeks of the 3-month study visit were excluded from this analysis. Figure 1 shows Bland Altman plots and graphs of the distribution of percentage variability for several of the biomarkers with the largest differences between COPD and controls (see Additional File 3 for the remaining biomarkers). Repeatability was assessed by calculating the proportion of values at three months that were within 25% of the baseline value (Table 5). Twenty-five percent was chosen as this reflects the typical total error associated with the types of 'research-grade' immunoassays used for this study [15]. Fibrinogen was the least variable biomarker with 3-month values that were within 25% of the baseline value for 89% of the COPD subjects. CRP was the most variable biomarker with only 21% of COPD subjects having a 3-month value that was within 25% of the baseline value. For most of the other analytes, the 3-month values were within 25% of the baseline values for 40-50% of the subjects. The same analysis was retrospectively applied to the data from the biomarker cohort for CC-16 and SP-D [7,8]. Ninety percent of CC-16 values at 3-months were within 25% of baseline and 74% of 3-month SP-D values were within 25% of baseline for the COPD subjects.

**Table 5:** Variability of selected biomarkers in stable subjects.

Biomarker	COPD Subjects*	Smoker Controls	Non-smoker Controls	All Subjects
CC-16	132/146 (90%)	29/35 (83%)	31/34 (91%)	192/215 (89%)

C-reactive protein	32/153 (21%)	7/36 (19%)	12/35 (34%)	51/224 (23%)

CCL24	51/144 (35%)	8/32 (25%)	16/35 (46%)	75/211 (36%)

Fibrinogen	139/156 (89%)	31/36 (86%)	35/36 (97%)	205/228 (90%)

Hepatocyte growth factor	57/152 (38%)	17/36 (47%)	19/36 (53%)	93/224 (42%)

Interleukin-6	55/147 (37%)	22/34 (65%)	24/36 (67%)	101/217 (47%)

Interleukin-8	67/141 (48%)	12/35 (34%)	13/33 (39%)	92/209 (44%)

Interleukin 12p40	64/124 (52%)	14/27 (52%)	20/30 (67%)	98/181 (54%)

CXCL10	86/152 (57%)	19/35 (54%)	23/34 (68%)	128/221 (58%)

CCL2	72/154 (47%)	22/36 (61%)	13/36 (36%)	107/226 (47%)

Matrix metalloproteinase-9	53/154 (34%)	8/36 (22%)	18/36 (50%)	79/226 (35%)

CCL23	93/154 (60%)	20/36 (56%)	23/36 (64%)	136/226 (60%)

Myeloperoxidase	52/154 (34%)	7/35 (20%)	15/36 (42%)	74/225 (33%)

CCL-18	53/135 (39%)	15/26 (58%)	15/35 (43%)	83/196 (42%)

Prolactin	47/148 (32%)	7/36 (19%)	9/35 (26%)	63/219 (29%)

Surfactant Protein D	107/145 (74%)	26/35 (74%)	30/34 (88%)	163/214 (76%)

Expressed as a percentage of subjects in which the 3-month value was within 25% of the baseline value. *COPD subjects with exacerbations within 30 days of the 3-month visit or subjects using oral steroids within 14 days of the 3 month visit were excluded.

### Correlation of biomarkers with the severity of COPD and the presence of emphysema

For the baseline assessments, six biomarkers showed weak correlation with COPD disease severity as defined by GOLD stage: β-defensin-2 (r = 0.2, p = 0.005), IL-8 (r = 0.15, p = 0.043), MMP-8 (r = 0.19, p = 0.007), MMP-9 (r = 0.16, p = 0.024), adiponectin (r = 0.18, p = 0.009) and hepatocyte growth factor (r = -0.21, p = 0.003). MMP-8 (r = 0.15, p = 0.043), MMP-9 (r = 0.18, p = 0.011) and adiponectin (r = 0.26, p =< 0.001) also correlated weakly with the percentage of emphysema as defined by low attenuation area on chest CT scans; MMP-9 correlated with the radiologists' score of emphysema (r = 0.18, p = 0.010).

### Fibrinogen as a promising biomarker for COPD

The most stable biomarker over 3 months in the ECLIPSE biomarker cohort was fibrinogen. It was significantly elevated in COPD patients relative to both ex-smoking and non-smoking controls although it was not associated with the severity of COPD as assessed by GOLD score or the severity of emphysema. There was a weak association with the number of reported exacerbations (0, 1, 2, 3+) in the year prior to the study, with a Spearman correlation coefficient (r) of 0.21 (p = 0.003) and fibrinogen was elevated in those subjects with unresolved exacerbations at 3 months compared with subjects that did not exacerbate or whose exacerbations had resolved. Decreased exercise tolerance as measured by 6-minute walk distance was also associated with higher levels of plasma fibrinogen (r = -0.23, p = 0.001) and there were associations with BODE index (r = 0.20, p = 0.007) and MRC dyspnoea score (r = 0.17, p = 0.021).

## Discussion

A subgroup of the ECLIPSE cohort was used to assess thirty-four putative blood biomarkers for their associations with important characteristics of COPD. The analytes that were chosen have previously been associated with either local or systemic features of COPD [5,10]. In subjects with stable COPD, fifteen analytes had significantly different concentrations in the COPD cohort as compared with smoker and non-smoker controls. Twelve of these were assessed for repeatability; this revealed wide variation over 3 months for many of the analytes, particularly CRP. Similar variability in a number of the biomarkers assessed in the ECLIPSE cohort has been shown by Man and colleagues in a cohort of 41 COPD subjects over a two week period [16] though less variable results were reported when measured at a yearly interval [17].

It is known that COPD is an inflammatory disease [18] and indeed CRP, fibrinogen and IL-6, all considered to be markers of systemic inflammation, are all significantly raised in COPD subjects compared to control subjects in this and in other studies [19-22]. They have received much attention to determine their value as potential COPD biomarkers [23]. In addition, CRP, fibrinogen, and IL-6 have been reported to be elevated during exacerbations of COPD [24-28]. Increased CRP and fibrinogen have also been associated with increased risk for COPD hospitalizations [29-31]. In the ECLIPSE biomarker cohort, fibrinogen was elevated in COPD subjects, demonstrated excellent repeatability over time in stable disease and was associated with BODE index and MRC dyspnoea score. Data from this cohort have not confirmed a previously recorded correlation with GOLD stage [32], but baseline fibrinogen associates with exacerbation frequency in ECLIPSE and other cohorts [11,33] and with exercise tolerance. Thus it may reflect a more global assessment of disease severity and therefore be a useful biomarker in COPD. IL-6 and CRP are both significantly raised in COPD and are yet further raised during exacerbations; however they display wide variability in stable subjects over three months and therefore their value as useful biomarkers of COPD would appear to be limited. Additionally, we found no significant association between TNF-α and COPD despite an association being described previously [34]. Importantly, circulating TNF-α was detectable in only approximately 10% of the subjects in the ECLIPSE biomarker cohort. The finding of low circulating TNF-α and the inability to detect it in many subjects in the current study is similar to that reported for COPD subjects included in a study of the effects of infliximab [35]. Further evaluation of serum TNF-α in the full ECLIPSE cohort confirmed that it is not measurable in a large percentage of subjects [36]. We also observed that serum concentrations of SP-D were both reproducible and elevated in COPD subjects with unresolved exacerbations compared with those with stable disease. This finding is consistent with our earlier report associating baseline serum concentrations of SP-D with exacerbations in the first year of ECLIPSE [8] and with the findings of others [37,38].

Many of the analytes studied that were different between cases and controls may play important roles as chemoattractants for monocytes/macrophages (IL-8, IL-12p40, CCL2), lymphocytes (CCL18, CXCL10) and neutrophils (IL-8) in driving inflammation in individuals with COPD. Unfortunately, many of the analytes we assessed showed poor repeatability when measured at a 3-month interval and thus they may have limited utility as markers of disease progression or measures of the effects of drug intervention. It is however possible that they may be useful to help define subgroups of COPD subjects based on common underlying disease mechanisms [39]. Studies with larger numbers of subjects will be required to determine if this is the case.

Aberrant tissue destruction and repair is known to be central to the development of emphysema [10]. MMP-8 and MMP-9 are two proteases that are believed to play an important role in the tissue destruction that results in emphysema [40,41] and both are raised in COPD subjects in the biomarker cohort. Consistent with other studies [42,43], there was evidence that they correlated with GOLD stage and amount of emphysema and so may be useful in differentiating individuals with emphysema from those with predominantly airway disease. Hepatocyte growth factor plays a role in tissue regeneration [44] and was shown in a small study to be increased in bronchoalveolar lavage of COPD subjects and smokers with normal lung function [45]. The repeatability of MMP-8 remains untested; however the wide variability of MMP-9 and hepatocyte growth factor over 3 months suggests that they are unlikely to be useful in clinical applications.

β-defensin-2 and myeloperoxidase play a role in pulmonary immunity [46,47]. We provide supportive evidence that circulating myeloperoxidase is raised in COPD [48] and show that β-defensin-2 is also higher in individuals with this disease. β-defensin-2 may also reflect disease severity as defined by GOLD stage. However additional profiling is required before it can be confidently deemed a useful biomarker in COPD.

Adiponectin and prolactin levels have been shown to be altered in subjects with COPD [5,49], however the mechanisms underlying this remain uncertain. Both have wide ranging metabolic effects and possible roles in the inflammatory response [50]. For both analytes, significant differences were seen between COPD subjects and only one of the control groups and marked variability was seen in stable disease. Although highly elevated in COPD subjects, the temporal variability associated with serum myeloperoxidase may confound its use in clinical trials. However, adiponectin, prolactin and myeloperoxidase may have some utility in diagnostic tests that combine a panel of inflammatory markers to describe the underlying inflammatory process in some COPD patients.

Our study has several limitations. In assessing putative biomarkers for repeatability, measures were taken at baseline and at 3 months which is the approximate duration of many COPD Phase IIa intervention studies. Though this interval is relatively short, it is conceivable that during this time period there may have been significant clinical changes, changes in pharmacological treatment or as we observed in our cohort, intercurrent exacerbations. Any of these could create a "true" change in biomarker level and thus the assessment of repeatability may overestimate how variable markers are over time. For example, Hurst [24], reported that the concentrations of CCL18, adiponectin, CRP, and IL-6 are increased during exacerbations. In order to better estimate reproducibility during stable disease, results from subjects with exacerbations that were not resolved at least 30 days prior to the 3 month visit were excluded from the repeatability analysis as were subjects who had been taking oral steroids within 2 weeks of the 3 month visit. The control populations in the ECLIPSE cohort were chosen on the basis of smoking status and the absence of COPD. Individuals may have co-morbidities that are inflammatory in nature and therefore influence the levels of biomarkers. However this is unlikely as details of co-morbidities were obtained at recruitment to the study. Though many of the biomarkers examined showed statistically significant differences between groups, no allowances were made for multiple comparisons in interpreting the statistical significance of the results [51]. Additionally corrections had to be made for differences in baseline characteristics between cases and controls as this study was intended to be exploratory with the primary intent of identifying biomarkers for follow up in the full ECLIPSE cohort or other COPD cohorts.

## Conclusions

We have identified several potentially clinically relevant blood biomarkers that are elevated in COPD subjects when compared to controls. In particular, fibrinogen appears to be the most repeatable over the three-month period tested. Surfactant protein D and CC-16 have also been shown to be repeatable [7,8]. These now need to be more rigorously assessed to clarify their repeatability in stable disease and studied in a longitudinal fashion to ascertain whether they can be used as surrogates for disease phenotype, severity and/or progression. Interventional studies will also be needed to qualify them for use as intermediates in COPD drug development.

## Competing interests

JD has no competing interests. EKS received grant support and consulting fees from GlaxoSmithKline for studies of COPD genetics, and he received honoraria and consulting fees from AstraZeneca. BEM, LDE and RTS are employees of GlaxoSmithKline (GSK) and own stock and stock options in GSK. DAL has received consultancy fees, honoraria and grant funding from GlaxoSmithKline.

## Authors' contributions

All authors participated in data analysis and interpretation of the results. JD drafted the manuscript. All authors read, provided input for, and approved the final manuscript.

## Supplementary Material

Additional file 1 **Additional Table **1. Biomarker Assay Performance Information. Intra- and Inter-assay variability of biomarker assays. Click here for file

Additional file 2 **Additional Table **2. Biomarker results at 3 months for COPD subjects with an exacerbation within 30 days of the study visit and COPD subjects with no exacerbations or resolved exacerbations. Results for the biomarkers not shown in Table 4 in the main manuscript. Click here for file

Additional file 3 **Additional Figure **1. Bland Altman plots and frequency histograms of differences between baseline and 3-month results illustrating biomarker variability. Results for the biomarkers not shown in Figure 1 within the main manuscript. Click here for file
