# Supplementary material for: COPD association and repeatability of blood biomarkers in the ECLIPSE cohort
Source: Respir Res. 2011 Nov 4;12(1):146. doi: 10.1186/1465-9921-12-146 (PMC3247194; doi:10.1186/1465-9921-12-146)
Supplement: Additional file 1 — Additional Table 1. Biomarker Assay Performance Information. Intra- and Inter-assay variability of biomarker assays. [file 1465-9921-12-146-S1.PDF]

Additional Table 1. Biomarker Assay Performance Information

		Intra-Assay Variability		Inter-Assay Variability	
**Biomarker**	**LLOQ***	**%RE**	**%CV**	**%RE**	**%CV**
Adiponectin	1.6 ng/mL	-4.4	6.4	-5.1	8.7
β-defensin-2	5.5 pg/mL	n.d.	10.2	0.3	16.6
Brain-derived neurotrophic growth factor	155 pg/mL	14.9	7.5	n.d.	7.4
C-reactive protein	5.9 pg/mL	-13.0	7.6	n.d.	4.5
CCL2	1.6 pg/mL	-8.3	11.8	n.d.	8.5
CCL4	1.6 pg/mL	-11.9	9.7	n.d.	11.2
CCL18	1.0 ng/mL	6.0	10.9	5.0	6.8
CCL23	2.4 pg/mL	7.4	10.5	n.d.	13.7
CCL24	30.0 pg/mL	16.1	13.3	n.d.	9.0
CXCL5	5.8 pg/mL	-6.9	18.5	n.d.	19.6
CXCL7	6.25 pg/mL	n.d.	9.1	8.3	16.0
CXCL10	5.4 pg/mL	-42.9	13.2	n.d	8.7
CXCL11	1.6 pg/mL	-20.1	18.3	n.d.	11.4
Fibrinogen	5.4 mg/dL	2.4	1.2	n.d.	1.9
Hepatocyte growth factor	1.6 pg/mL	-12.5	16.2	7.8	8.8
Interferon-γ	0.8 pg/mL	-20.6	12.5	n.d.	10.4
Interleukin-1 receptor antagonist	15.6 pg/mL	-28.2	10.5	n.d.	8.0
Interleukin-1β	0.4 pg/mL	5.77	18.9	n.d.	6.9
Interleukin-6	0.4 pg/mL	-9.77	15.5	n.d.	6.3
Interleukin-8	0.8 pg/mL	-24.37	15.4	n.d.	12.5
Interleukin-10	0.8 pg/mL	-21.97	9.3	n.d.	16.3
Interleukin-12p40	1.2 pg/mL	-28.23	11.9	n.d.	6.2
Interleukin-15	1.6 pg/mL	46.9	13.9	n.d.	6.2
Interleukin-17	1.6 pg/mL	-18.1	12.5	n.d.	8.4
Leptin	31.25 pg/mL	n.d.	12.2	4.8	12.8
MMP-8	2.0 ng/mL	2.8	15.7	2.0	10.4
MMP-9	40.8 pg/mL	-29.9	13.9	n.d.	11.6
Myeloperoxidase	3.9 pg/mL	n.d.	12.3	n.d.	12.5
Prolactin	500 pg/mL	n.d.	12.3	n.d.	7.8
Tissue inhibitor of metalloproteinase-1	24.5 ng/mL	18.3	15.0	n.d.	13.1
Transforming growth factor-α	4.8 pg/mL	11.4	7.2	n.d.	12.8
Tumor necrosis factor-α	4.7 pg/mL	-28.6	9.2	n.d.	8.0
Tumor necrosis factor receptor type I	4.6 pg/mL	-5.1	11.7	n.d.	10.8
Tumor necrosis factor receptor type II	45 pg/mL	-30.2	11.1	n.d.	10.8

*Lower limit of quantitation (LLOQ) at the minimum required dilution for sample testing. n.d.: not done
