# Supplementary material for: COPD association and repeatability of blood biomarkers in the ECLIPSE cohort
Source: Respir Res. 2011 Nov 4;12(1):146. doi: 10.1186/1465-9921-12-146 (PMC3247194; doi:10.1186/1465-9921-12-146)
Supplement: Additional file 2 — Additional Table 2. Biomarker results at 3 months for COPD subjects with an exacerbation within 30 days of the study visit and COPD subjects with no exacerbations or resolved exacerbations. Results for the biomarkers not shown in Table 4 in the main manuscript. [file 1465-9921-12-146-S2.PDF]

Additional Table 2. Biomarker results at 3 months for COPD subjects with an exacerbation within 30 days of the study visit and COPD subjects with no exacerbations or resolved exacerbations.

Biomarker	Exacerbations within 30 days		No or Resolved Exacerbations		p-value
	Median (IQR)	N	Median (IQR)	N	
Transforming growth factor-α (pg/mL)	10.0 (26.9)	33	3.4 (10.9)	151	0.045
Interleukin-17 (pg/mL)	0.8 (0.0)	33	0.8 (0.0)	154	0.125
Hepatocyte growth factor (pg/mL)	596.0 (635.9)	35	505.3 (396.2)	159	0.155
CCL2 (pg/mL)	650.0 (385.0)	33	625.0 (495.0)	154	0.370
Interleukin 1 beta (pg/mL)	0.2 (0.0)	33	0.2 (0.0)	152	0.402
CXCL10 (pg/mL)	77.8 (43.8)	33	75.3 (43.2)	154	0.433
Myeloperoxidase (pg/mL)	34140.0 (37110.0)	32	27720.0 (29720.0)	154	0.437
Matrix metalloproteinase-9 (pg/mL)	231040.0 (237740.0)	33	213140.0 (182700.0)	154	0.439
Interleukin-15 (pg/mL)	0.8 (0.0)	31	0.8 (0.0)	145	0.479
CCL24 (pg/mL)	90.0 (135.0)	33	94.5 (122.0)	145	0.498
Prolactin (pg/mL)	622.5 (340.0)	32	560.0 (517.5)	148	0.556
Tissue inhibitor of metalloproteinase-1 (pg/mL)	345000.0 (199500.0)	33	353850.0 (240900.0)	152	0.571
Brain-derived neurotrophic growth factor (pg/mL)	33360.0 (13720.0)	33	35440.0 (16700.0)	154	0.572
CCL18 (pg/mL)	105500.0 (79500.0)	32	101000.0 (73859.4)	139	0.586
CC-16 (ng/mL)	5.8 (2.8)	32	5.7 (3.9)	149	0.587
Tumor necrosis factor-α (pg/mL)	2.4 (0.0)	33	2.4 (0.0)	154	0.594
CXCL11 (pg/mL)	22.0 (19.2)	33	21.4 (17.4)	154	0.736
Tumor necrosis factor receptor type I (pg/mL)	1775.0 (695.0)	33	1641.1 (1325.0)	154	0.787
Interleukin-12p40 (pg/mL)	1.6 (3.8)	31	1.8 (2.6)	143	0.808
Interleukin-8 (pg/mL)	8.1 (4.7)	32	8.4 (5.6)	149	0.818
CCL4 (pg/mL)	121.6 (73.2)	33	116.4 (76.0)	153	0.855
Interleukin-10 (pg/mL)	0.4 (0.0)	33	0.4 (0.0)	154	0.899
CCL23 (pg/mL)	511.0 (128.6)	33	516.2 (218.0)	154	0.922
Tumor necrosis factor receptor type II (pg/mL)	1205.0 (550.0)	32	1145.0 (645.0)	150	0.931
Interferon-γ (pg/mL)	0.4 (0.0)	33	0.4 (0.0)	152	0.932
Interleukin-1 receptor antagonist (pg/mL)	200.7 (175.1)	32	203.8 (244.6)	149	0.947

Biomarker	Exacerbations within 30 days		No or Resolved Exacerbations		p-value
CXCL5 (pg/mL)	1700.0 (1195.0)	33	1610.0 (1260.0)	154	0.981

Values are expressed as median (IQR), p values are based on log transformed results.

The table shows those biomarkers that were not significantly different between the two groups.
