# Supplementary material for: COPD association and repeatability of blood biomarkers in the ECLIPSE cohort
Source: Respir Res. 2011 Nov 4;12(1):146. doi: 10.1186/1465-9921-12-146 (PMC3247194; doi:10.1186/1465-9921-12-146)
Supplement: Additional file 3 — Additional Figure 1. Bland Altman plots and frequency histograms of differences between baseline and 3-month results illustrating biomarker variability. Results for the biomarkers not shown in Figure 1 within the main manuscript. [file 1465-9921-12-146-S3.PDF]

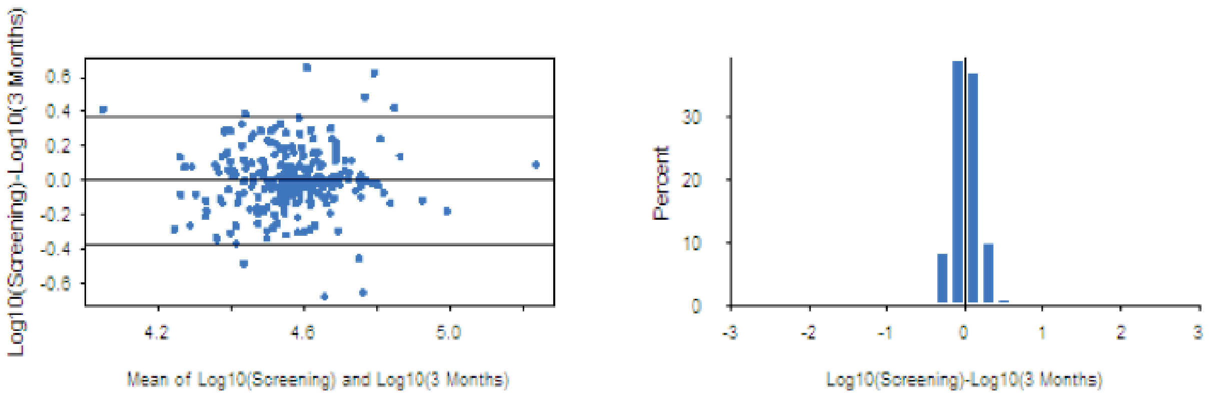

Brain derived neurotrophic growth factor

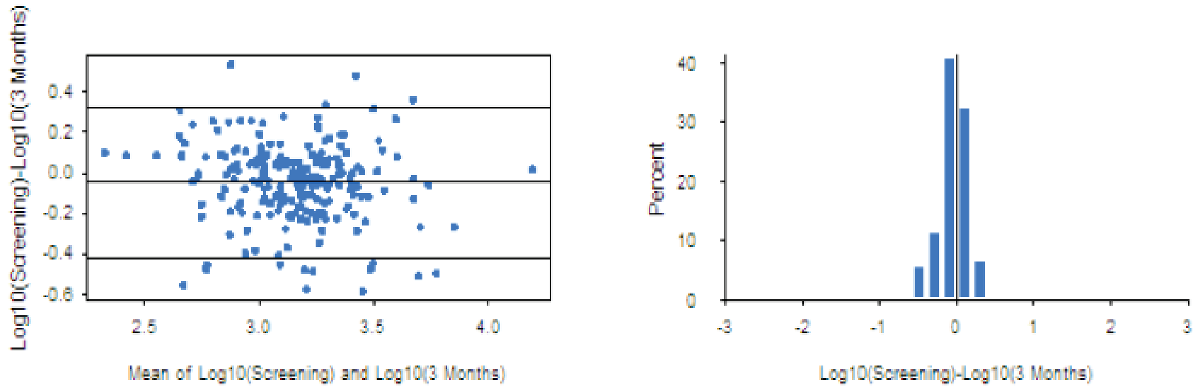

CXCL5

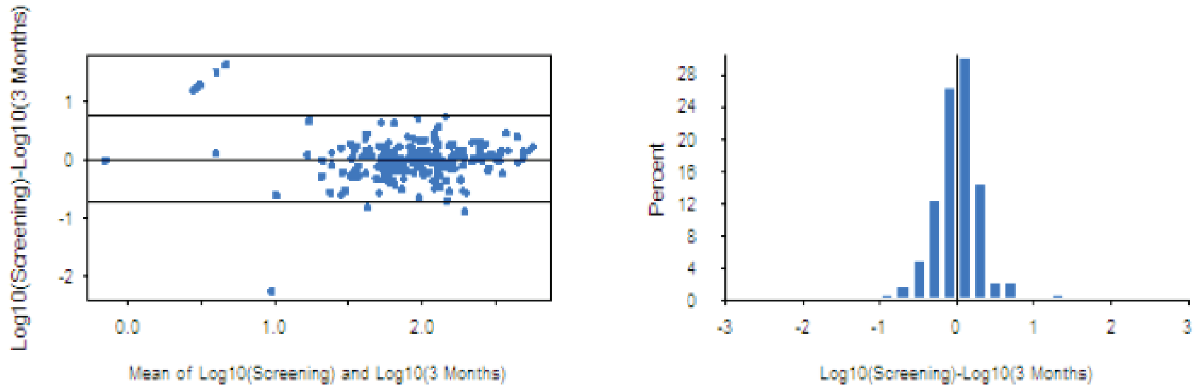

CCL24

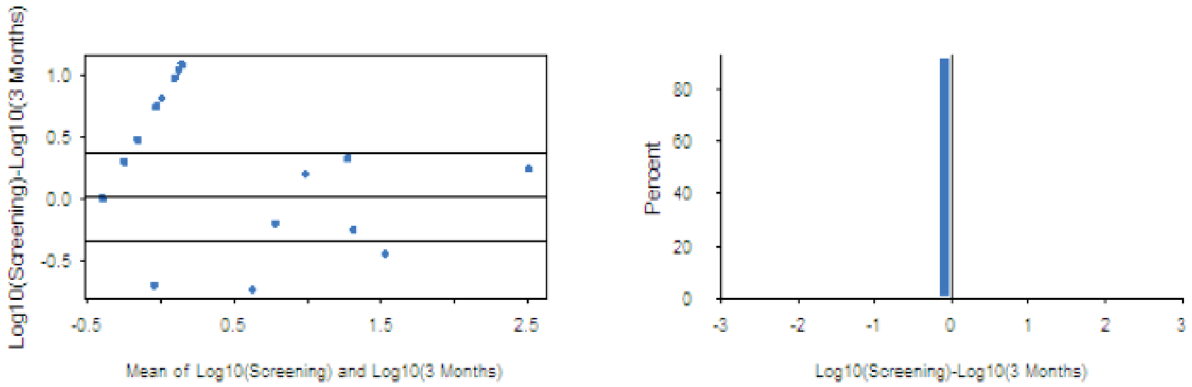

Interferon-γ

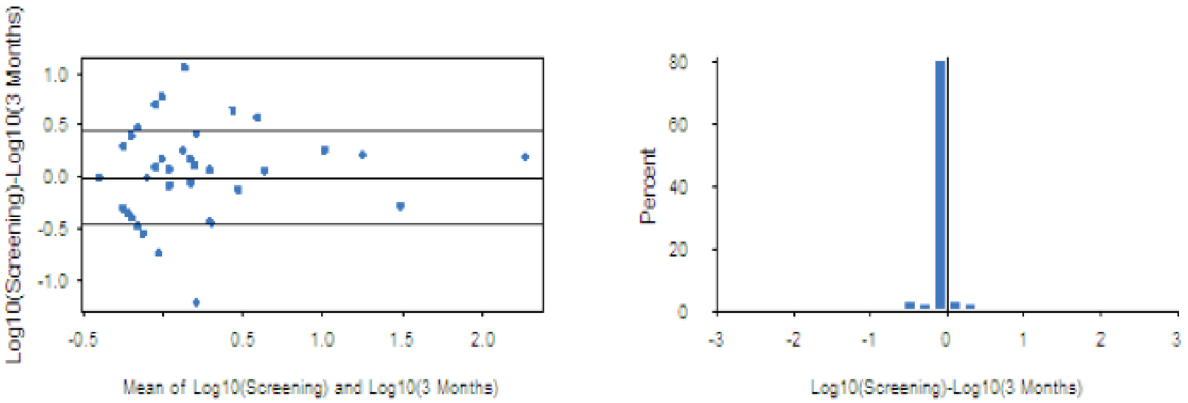

Interleukin-10

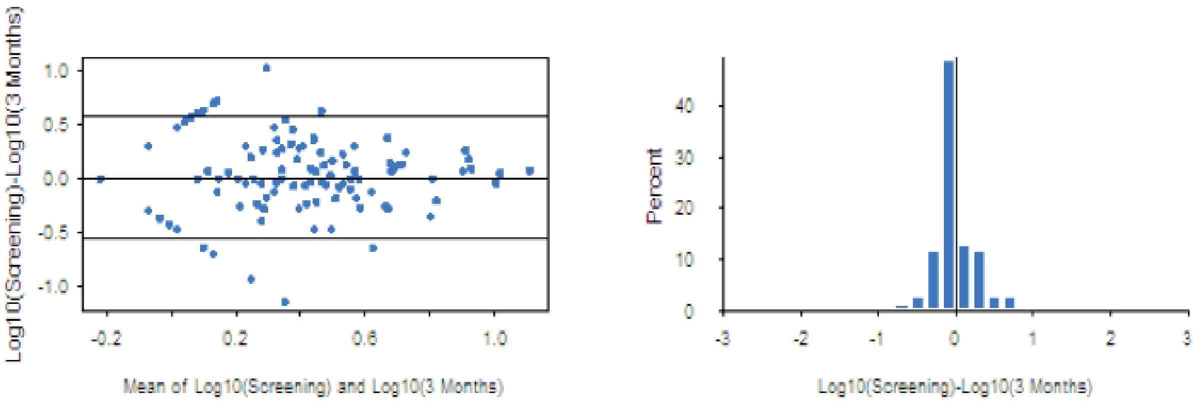

Interleukin-12p40

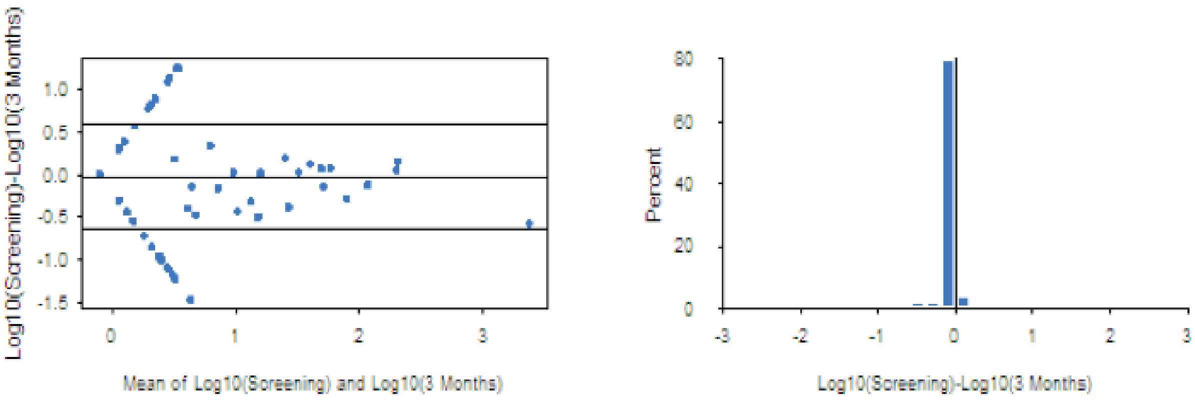

Interleukin-15

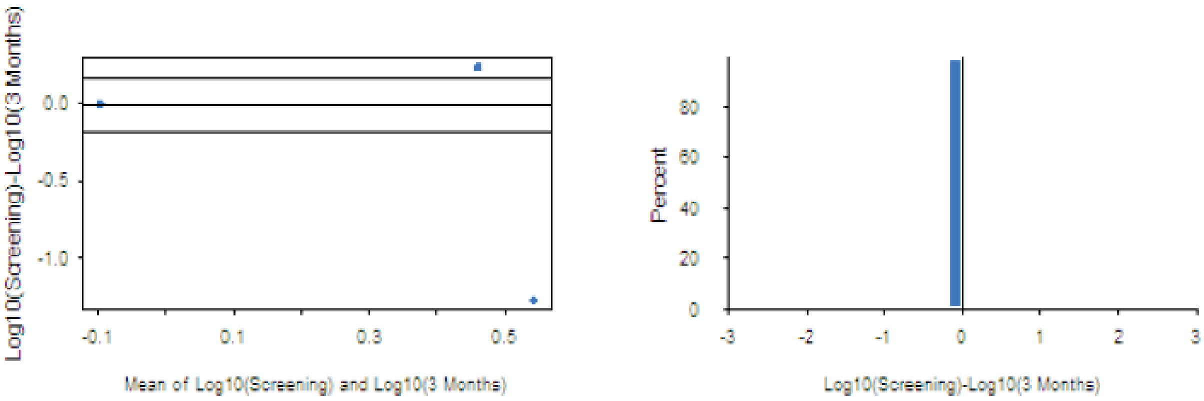

Interleukin-17

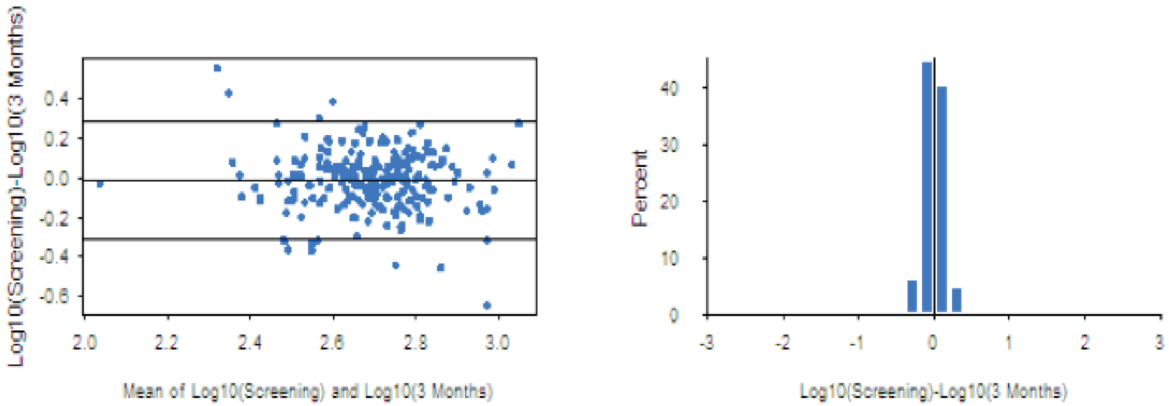

CCL23

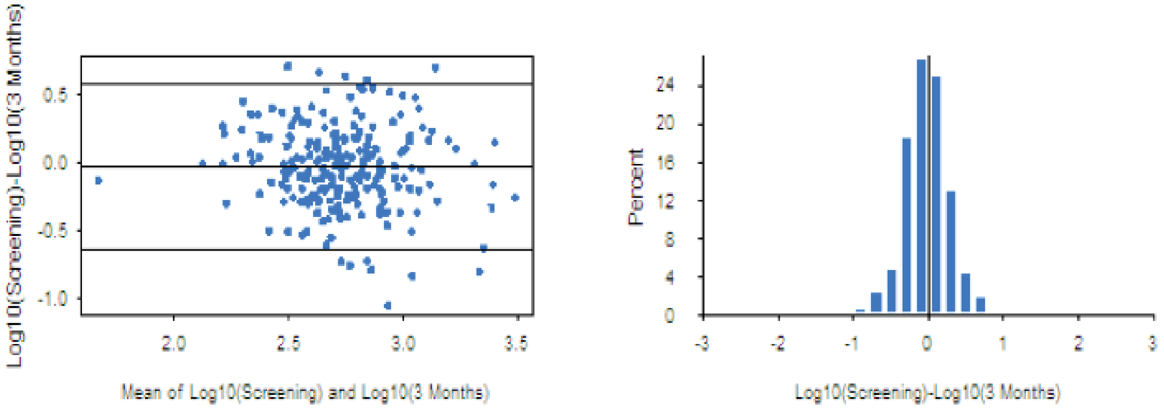

Prolactin

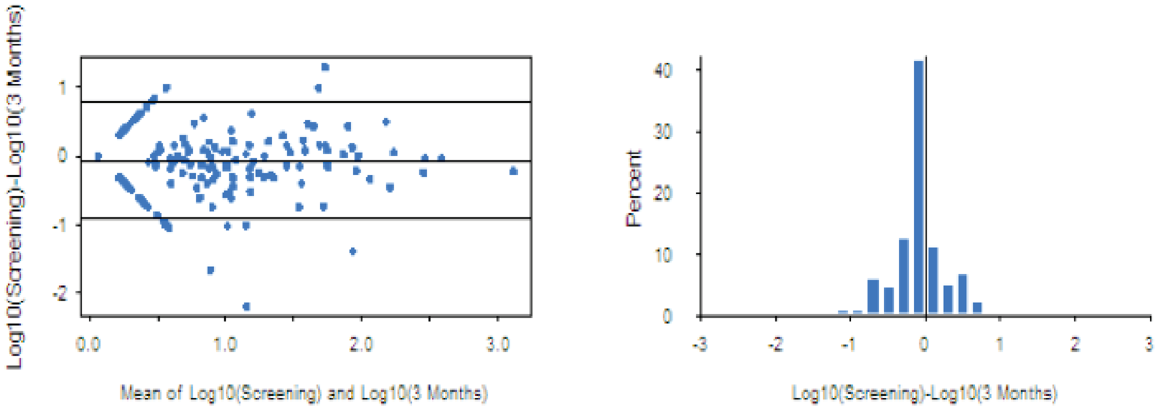

Transforming growth factor-α

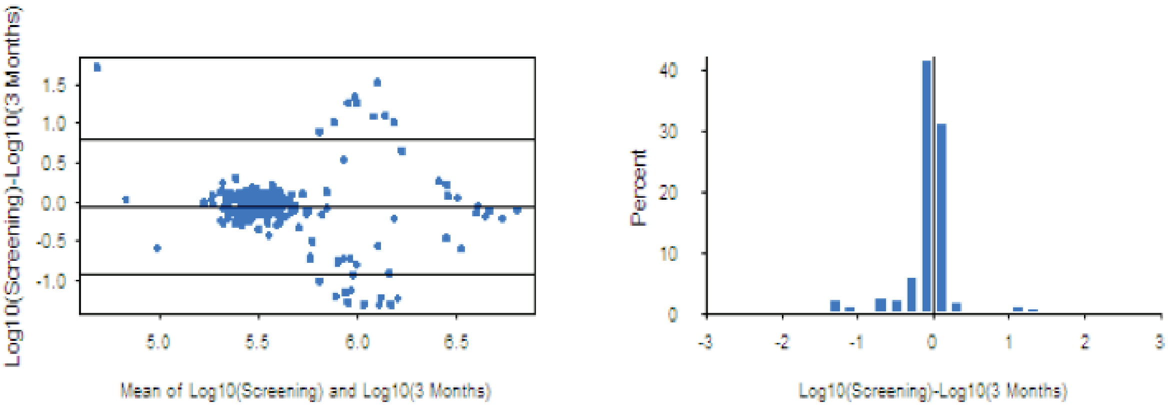

Tissue inhibitor of metalloproteinase-1

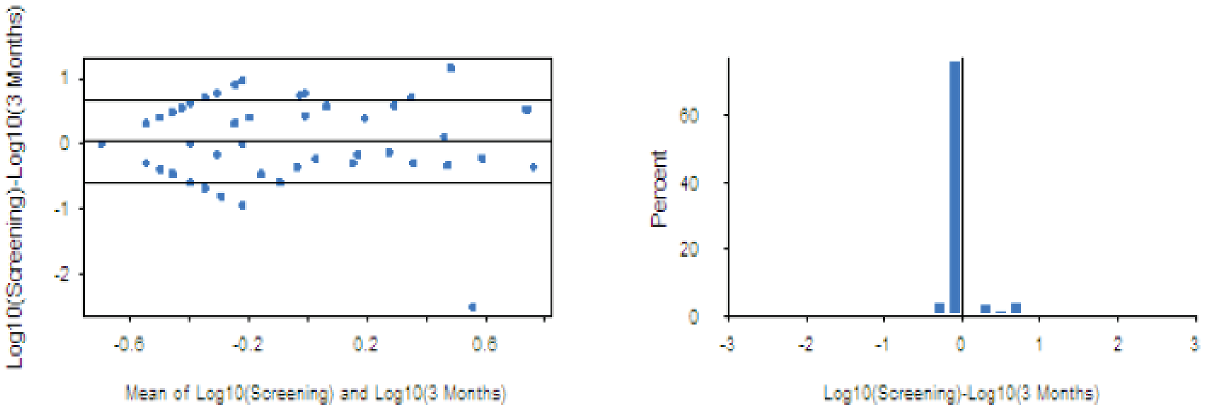

Interleukin-1B

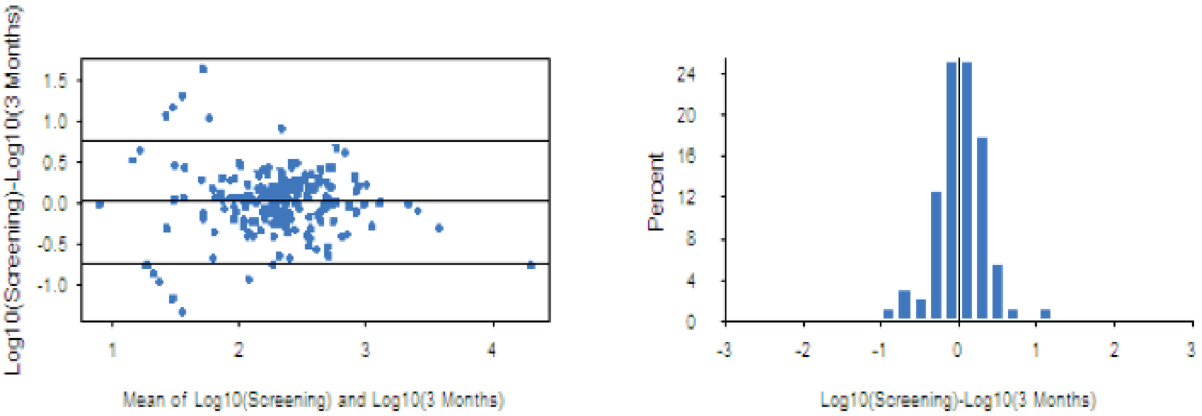

Interleukin-1 receptor antagonist

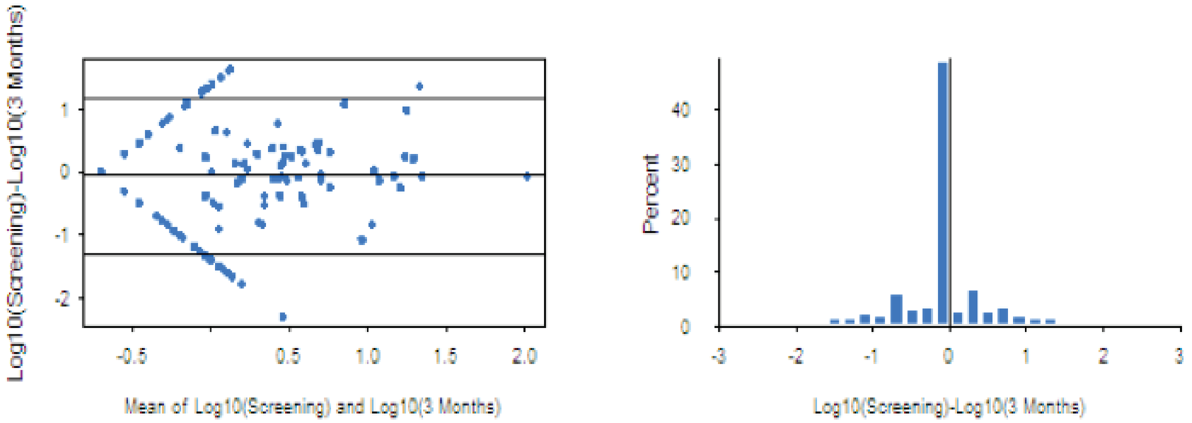

Interleukin-6

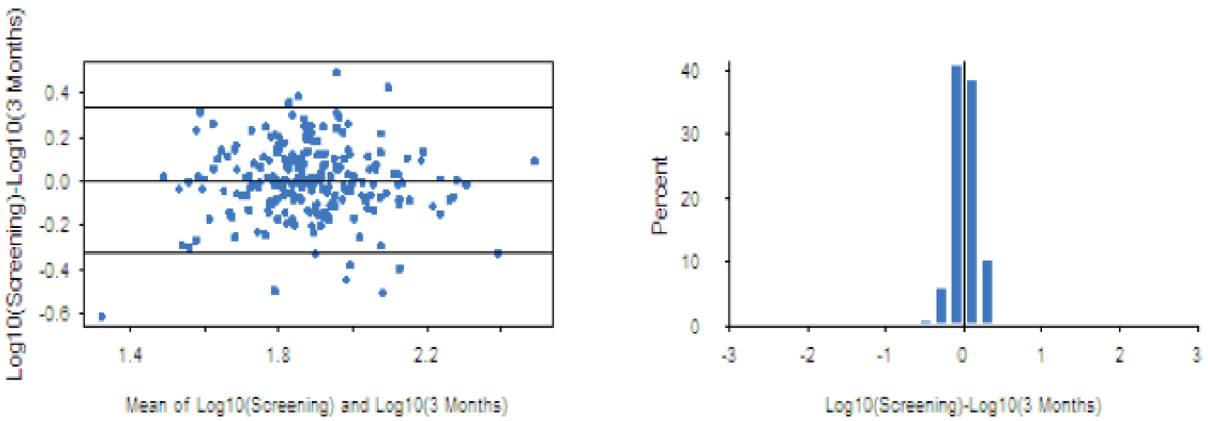

CXCL10

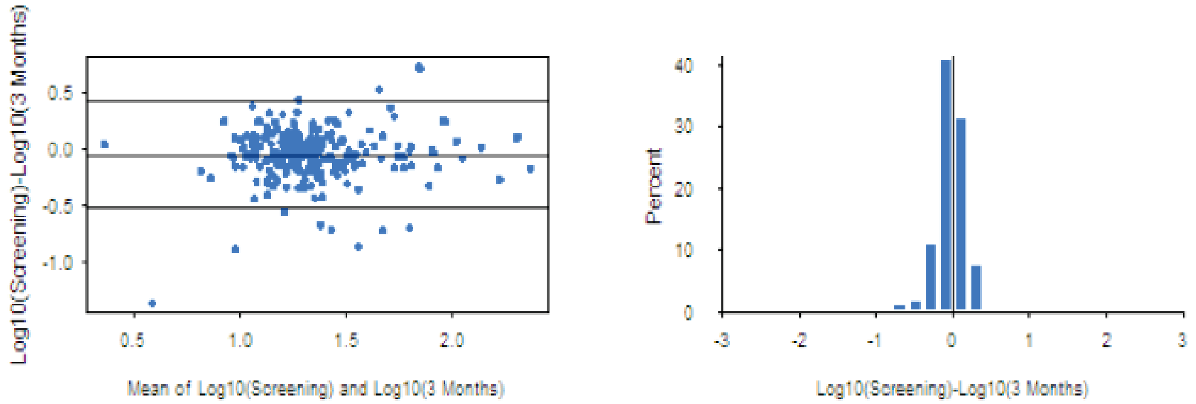

CXCL11

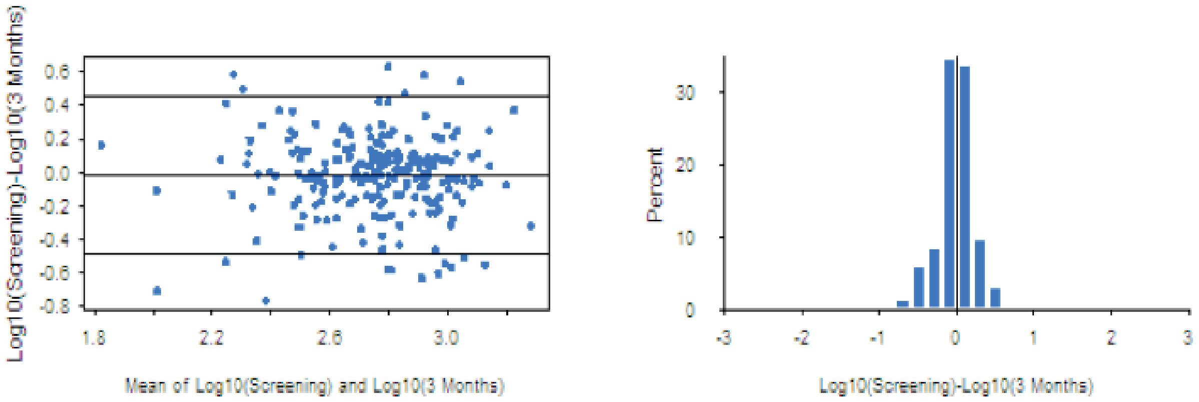

CCL2

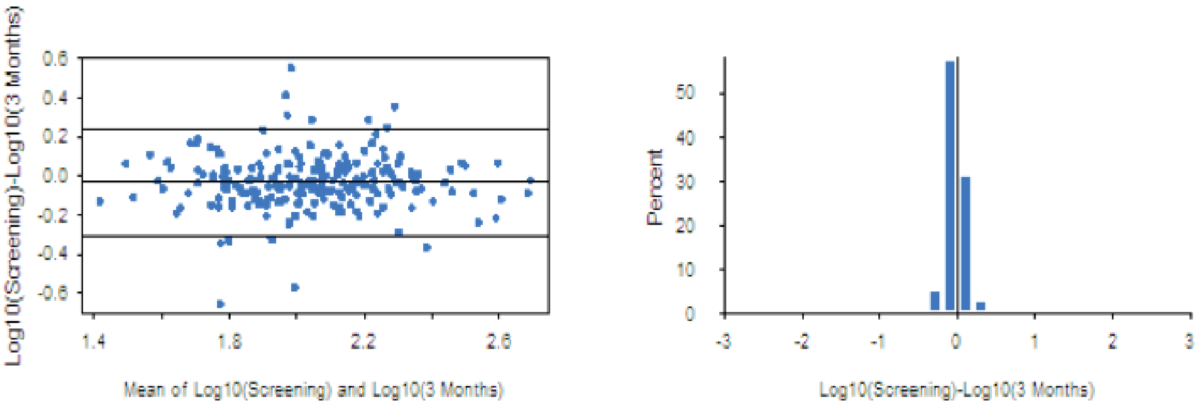

CCL4

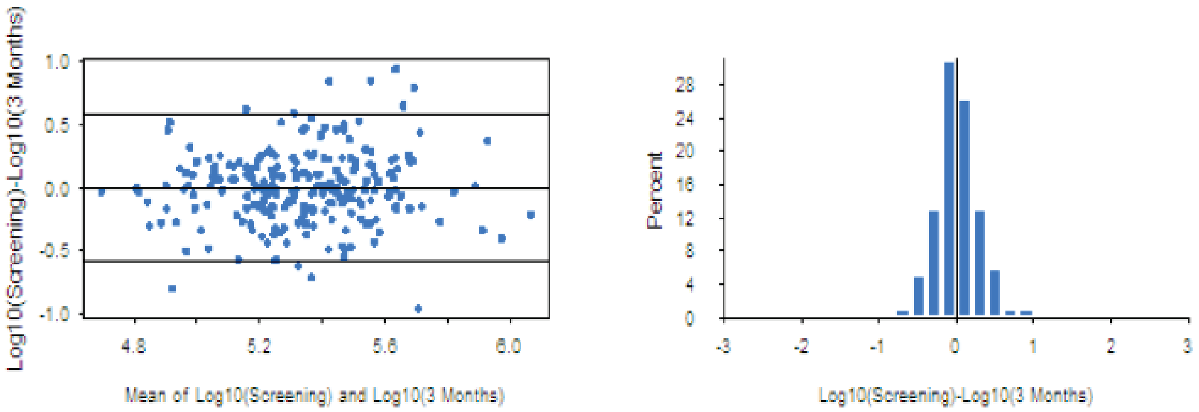

Matrix Metalloproteinase-9

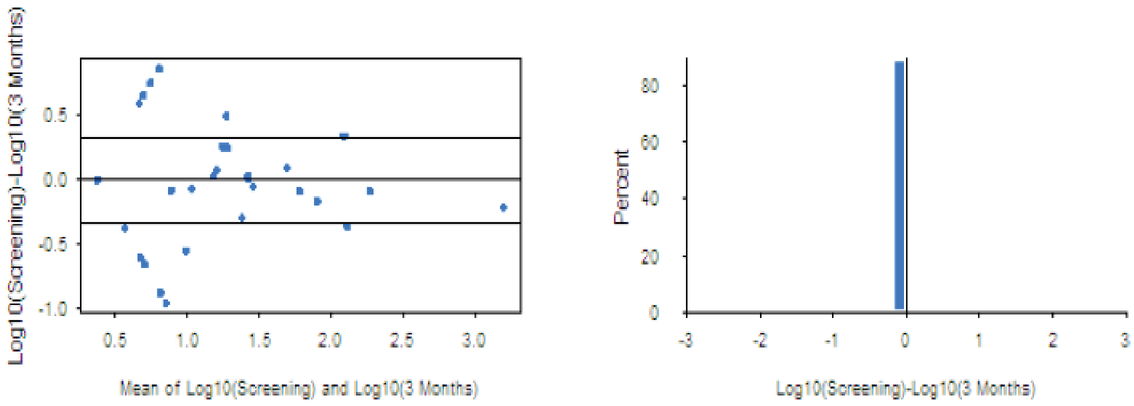

Tumor necrosis factor-α

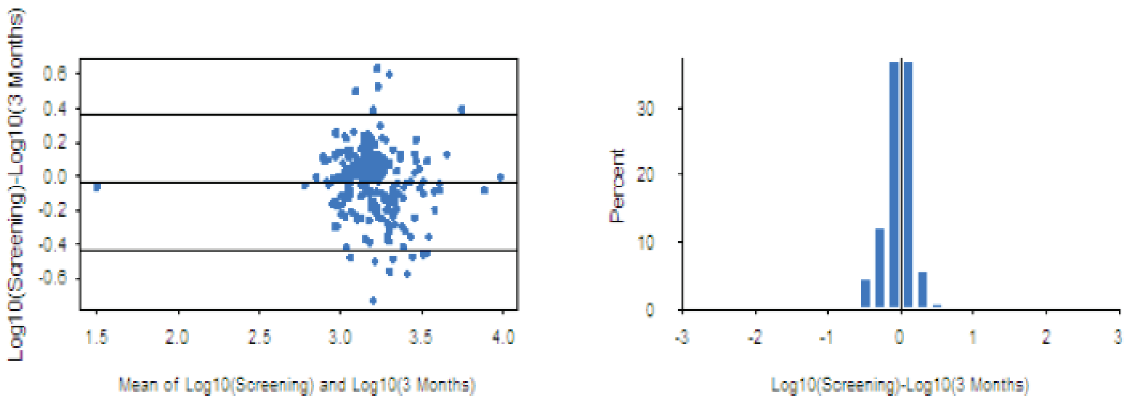

Tumor necrosis factor receptor type 1

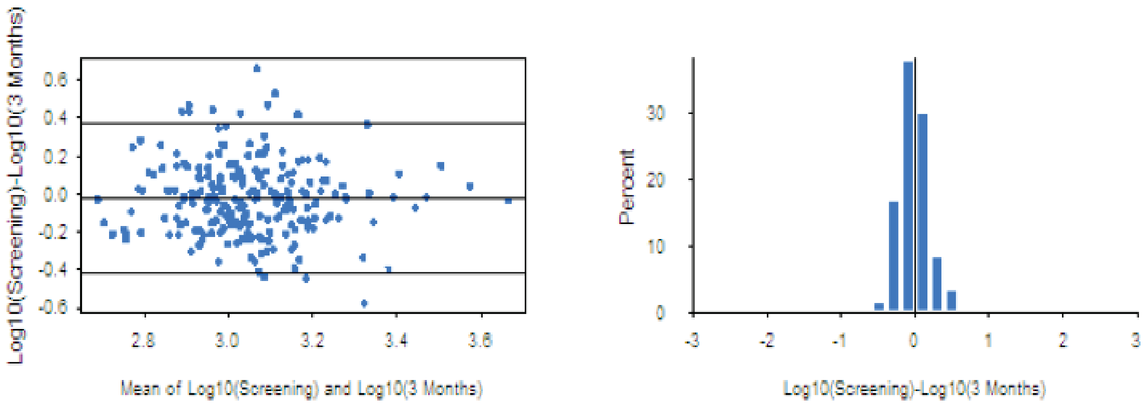

Tumor necrosis factor receptor type 2
